# Supplementary material for: Exploring the Intersection of ADHD and Music: A Systematic Review
Source: Behav Sci (Basel). 2025 Jan 13;15(1):65. doi: 10.3390/bs15010065 (PMC11762814; doi:10.3390/bs15010065)
Supplement: Supplementary file 1 [file behavsci-15-00065-s001.zip › behavsci-3317418-supplementary.pdf]

Supplementary Material

Table S1: PRISMA 2020 Checklist

	Item #	Checklist item	Location where item is reported
**TITLE**			
Title	1	Identify the report as a systematic review.	Title; page 1
**ABSTRACT**			
Abstract	2	See the PRISMA 2020 for Abstracts checklist.	Abstract; page 1
**INTRODUCTION**			
Rationale	3	Describe the rationale for the review in the context of existing knowledge.	Sections 1.1. to 1.4.; pages 1 to 4
Objectives	4	Provide an explicit statement of the objective(s) or question(s) the review addresses.	Section 1.5 “Aims and Objectives”; pages 4 and 5
**METHODS**			
Eligibility criteria	5	Specify the inclusion and exclusion criteria for the review and how studies were grouped for the syntheses.	Section 2.2 “Inclusion and exclusion criteria”; pages 5 and 6.
Information sources	6	Specify all databases, registers, websites, organisations, reference lists and other sources searched or consulted to identify studies. Specify the date when each source was last searched or consulted.	Section 2.1 “Search strategy”; page 5
Search strategy	7	Present the full search strategies for all databases, registers and websites, including any filters and limits used.	Section 2.1 “Search strategy”; page 5
Selection process	8	Specify the methods used to decide whether a study met the inclusion criteria of the review, including how many reviewers screened each record and each report retrieved, whether they worked independently, and if applicable, details of automation tools used in the process.	Section 2.3 “Screening” and section 2.4 “2.4 Data extraction”; page 6
Data collection process	9	Specify the methods used to collect data from reports, including how many reviewers collected data from each report, whether they worked independently, any processes for obtaining or confirming data from study investigators, and if applicable, details of automation tools used in the process.	Section 2.4 “2.4 Data extraction”; page 6
Data items	10a	List and define all outcomes for which data were sought. Specify whether all results that were compatible with each outcome domain in each study were sought (e.g. for all measures, time points, analyses), and if not, the methods used to decide which results to collect.	Section 2.5 “Type of data extracted”; page 6
	10b	List and define all other variables for which data were sought (e.g. participant and intervention characteristics, funding sources). Describe any assumptions made about any missing or unclear information.	Section 2.5 “Type of data extracted”; page 6
Study risk of bias assessment	11	Specify the methods used to assess risk of bias in the included studies, including details of the tool(s) used, how many reviewers assessed each study and whether they worked independently, and if applicable, details of automation tools used in the process.	Section 2.4 “2.4 Data extraction”; page 6. The risk of bias was not assessed; see section 4.3 “Limitations” page 25
Effect measures	12	Specify for each outcome the effect measure(s) (e.g. risk ratio, mean difference) used in the synthesis or presentation of results.	Effect measures such as risk ratios and mean differences for each outcome were not assessed; see section 4.3 “Limitations” page 25
Synthesis methods	13a	Describe the processes used to decide which studies were eligible for each synthesis (e.g. tabulating the study intervention characteristics and comparing	Section 2.7 “Analysis”; page 6. Tables 1-3; pages 9 to 17

	Item #	Checklist item	Location where item is reported
		against the planned groups for each synthesis (item #5)).	
	13b	Describe any methods required to prepare the data for presentation or synthesis, such as handling of missing summary statistics, or data conversions.	Section 2.7 “Analysis”; page 6. The guidelines for narrative synthesis proposed by Popay et al. were followed (Popay et al., 2006)
	13c	Describe any methods used to tabulate or visually display results of individual studies and syntheses.	Tables 1-3; pages 9 to 17
	13d	Describe any methods used to synthesize results and provide a rationale for the choice(s). If meta-analysis was performed, describe the model(s), method(s) to identify the presence and extent of statistical heterogeneity, and software package(s) used.	Section 2.7 “Analysis”; page 6. The guidelines for narrative synthesis proposed by Popay et al. were followed (Popay et al., 2006)
	13e	Describe any methods used to explore possible causes of heterogeneity among study results (e.g. subgroup analysis, meta-regression).	We were not able to perform subgroup analyses or meta-regressions; see section 4.3 “Limitations” page 25
	13f	Describe any sensitivity analyses conducted to assess robustness of the synthesized results.	We were not able to perform sensitivity analyses; see section 4.3 “Limitations” page 25
Reporting bias assessment	14	Describe any methods used to assess risk of bias due to missing results in a synthesis (arising from reporting biases).	Section 2.4 “2.4 Data extraction”; page 6. We used the Joanna Briggs Insti-tute (JBI) Critical Appraisal Tools for randomized controlled trials and qua-si-experimental studies (Joanna Briggs Institute, 2017); see supplementary S2.
Certainty assessment	15	Describe any methods used to assess certainty (or confidence) in the body of evidence for an outcome.	Section 2.4 “2.4 Data extraction”; page 6. We used the Joanna Briggs Insti-tute (JBI) Critical Appraisal Tools for randomized controlled trials and qua-si-experimental studies (Joanna Briggs Institute, 2017); see supplementary S2.
**RESULTS**			
Study selection	16a	Describe the results of the search and selection process, from the number of records identified in the search to the number of studies included in the review, ideally using a flow diagram.	Section 3.1 “Search results”; page 7 and PRISMA flow diagram; page 8
	16b	Cite studies that might appear to meet the inclusion criteria, but which were excluded, and explain why they were excluded.	PRISMA flow diagram; page 8
Study characteristics	17	Cite each included study and present its characteristics.	Section 3.2 “Study Characteristics”; page 7
Risk of bias in studies	18	Present assessments of risk of bias for each included study.	We used the Joanna Briggs Institute (JBI) Critical Appraisal Tools for randomized controlled trials and quasi-experimental studies (Joanna Briggs Institute, 2017); see supplementary S2. See also section 4.3 “Limitations” page 25
Results of individual studies	19	For all outcomes, present, for each study: (a) summary statistics for each group (where appropriate) and (b) an effect estimate and its precision (e.g. confidence/credible interval), ideally using structured tables or plots.	Tables 1-3; pages 9 to 17
Results of syntheses	20a	For each synthesis, briefly summarise the characteristics and risk of bias among contributing studies.	The risk of bias was not assessed; see section 4.3 “Limitations” page 25
	20b	Present results of all statistical syntheses conducted. If meta-analysis was done, present for each the summary estimate and its precision (e.g. confidence/credible interval) and measures of statistical heterogeneity. If comparing groups, describe the direction of the effect.	Tables 1-3; pages 9 to 17
	20c	Present results of all investigations of possible causes of heterogeneity among study results.	Section 4.3 “Limitations” page 25

	Item #	Checklist item	Location where item is reported
	20d	Present results of all sensitivity analyses conducted to assess the robustness of the synthesized results.	We were not able to perform sensitivity analyses; see section 4.3 “Limitations” page 25
Reporting biases	21	Present assessments of risk of bias due to missing results (arising from reporting biases) for each synthesis assessed.	Section 4.3 “Limitations” page 25
Certainty of evidence	22	Present assessments of certainty (or confidence) in the body of evidence for each outcome assessed.	Tables 1-3; pages 9 to 17. Section 4.3 “Limitations” page 25
**DISCUSSION**			
Discussion	23a	Provide a general interpretation of the results in the context of other evidence.	Section 4.1 “Summary of Results”, pages 20 to 23 and section 4.2 “Comparison with the Results of Previous Systematic Reviews”; page 23
	23b	Discuss any limitations of the evidence included in the review.	Section 4.3 “Limitations”; pages 23 to 25
	23c	Discuss any limitations of the review processes used.	Section 4.3 “Limitations”; pages 23 to 25
	23d	Discuss implications of the results for practice, policy, and future research.	Section 4.4 “Implications and Future Directions”; pages 25 to 26. Section 5. “Conclusion”; page 26
**OTHER INFORMATION**			
Registration and protocol	24a	Provide registration information for the review, including register name and registration number, or state that the review was not registered.	The review was not registered. See section 4.3 “Limitations” page 25
	24b	Indicate where the review protocol can be accessed, or state that a protocol was not prepared.	See above.
	24c	Describe and explain any amendments to information provided at registration or in the protocol.	See abobe.
Support	25	Describe sources of financial or non-financial support for the review, and the role of the funders or sponsors in the review.	See funding statement on page 27
Competing interests	26	Declare any competing interests of review authors.	See conflicts of interest statement on page 27
Availability of data, code and other materials	27	Report which of the following are publicly available and where they can be found: template data collection forms; data extracted from included studies; data used for all analyses; analytic code; any other materials used in the review.	The exact search strategy for PubMed, Web of Science, and PsychInfo (Ovid) is given in section 2.1 “Search strategy” on page 5. All extracted data are reported in Tables 1-3 on pages 9 to 17 and in the supplementary material.

From: Page MJ et al. (2021).

Table S2: Quality assessment of included studies using the Joanna Briggs Institute (JBI) Critical Appraisal Tool for quasi-experimental studies (Joanna Briggs Institute, 2025)**.**

Study	Is it clear in the study what is the "cause" and what is the "effect"?	Was there a control group?	Were participants included in any comparisons similar?	Were the participants included in any comparisons receiving similar treatment/care, other than the exposure or intervention of interest?	Were there multiple measurements of the outcome, both pre and post the intervention/exposure?	Were the outcomes of participants included in any comparisons measured in the same way?	Were outcomes measured in a reliable way?	Was follow-up complete and if not, were differences between groups in terms of their follow-up adequately described and analysed?	Was appropriate statistical analysis used?
Abikoff et al., 1996	Yes	Yes	Yes	Yes	No	Yes	Yes	N/A	Yes
Aydinli et al., 2016	Yes	Yes	Yes	Yes	No	Yes	Yes	N/A	Yes
Cripe, 1986	Yes	No	Yes	Yes	Yes	Yes	Yes	N/A	Yes
Dong et al., 2022	Yes	No	Yes	Yes	Yes	Yes	Yes	N/A	Yes
Greenop & Kann, 2007	Yes	Yes	Yes	Yes	No	Yes	Yes	N/A	Yes
Madjar et al., 2020	Yes	Yes	Yes	Yes	Yes	Yes	Yes	N/A	Yes
Pelham et al., 2011- Exp.1	Yes	Yes	Yes	Yes	No	Yes	Yes	N/A	Yes
Pelham et al., 2011- Exp. 2	Yes	No	Yes	Yes	No	Yes	Yes	N/A	Yes

Sami et al., 2020	Yes	Yes	Yes	Yes	No	Yes	Yes	N/A	Yes
Windwer, 1981	No	No	Yes	Yes	Yes	Yes	Yes	N/A	Yes
Carrer, 2015	Yes	Yes	Unclear	Yes	No	Yes	Yes	N/A	Yes
Grob et al., 2023	Yes	Yes	Yes	Yes	No	Yes	Yes	N/A	Yes
Grob et al., 2022	Yes	Yes	Yes	Yes	No	Yes	Yes	N/A	Yes
Puyjarinet et al., 2017	Yes	No	Yes	Yes	No	Yes	Yes	N/A	Yes
Lee et al., 2024	Yes	No	Yes	Yes	No	Yes	Yes	Yes	Yes
Luo et al., 2023	Yes	Yes	Yes	Yes	Yes	Yes	Yes	N/A	Yes
Zemestani et al., 2023	Yes	No	Yes	Yes	Yes	Yes	Yes	Yes	Yes

Table S3: Quality assessment of included studies using the Joanna Briggs Institute (JBI) Critical Appraisal Tool for randomized controlled trials (Joanna Briggs Institute, 2025).

Study	Was true randomization used for assignment of participants to treatment groups?	Was allocation to treatment groups concealed?	Were treatment groups similar at the baseline?	Were participants blind to treatment assignment?	Were those delivering the treatment blind to treatment assignment?	Were treatment groups treated identically other than the intervention of interest?	Were outcome assessors blind to treatment assignment?	Were outcomes measured in the same way for treatment groups?	Were outcomes measured in a reliable way?	Was follow up complete and if not, were differences between groups in terms of their follow up adequately described and analysed?	Were participants analysed in the groups to which they were randomised?	Was appropriate statistical analysis used?	Was the trial design appropriate and any deviations from the standard RCT design (individual randomization, parallel groups) accounted for in the conduct and analysis of the trial?
Zimmermann et al., 2019	Yes	Yes	Yes	No	No	Yes	No	Yes	Yes	N/A	Yes	Yes	Yes
Park et al., 2023	Yes	Yes	Yes	No	No	Yes	Yes	Yes	Yes	Yes	Yes	Yes	Yes
Rickson et al., 2006	Yes	Yes	Yes	No	No	Yes	No	Yes	Yes	N/A	Yes	Yes	Yes
Rickson & Watkins, 2003	Yes	Yes	No	No	No	Yes	No	Yes	Yes	N/A	Yes	Yes	Yes
Zhu, 2022	Yes	Yes	Yes	No	No	Yes	Unclear	Yes	Yes	Yes	Yes	Yes	Yes
